# Options in the Treatment of Subacute Sclerosing Panencephalitis: Implications for Low Resource Areas

**DOI:** 10.1007/s11940-022-00710-x

**Published:** 2022-03-19

**Authors:** Pauline Samia, Katherine Oyieke, Dorcas Tunje, Anaita Udwadia-Hegde, Kristen Feemster, Ibrahim Oncel, Banu Anlar

**Affiliations:** 1 Department of Paediatrics and Child Health, Medical College, Aga Khan University, 3rd Parklands Avenue, P.O BOX 30270 00100, East Tower block, fourth floor Nairobi, Kenya; 2 Brain and Mind Institute, Aga Khan University, Nairobi, Kenya; 3 Department of Public Health and Primary Care, Ghent University, Ghent, Belgium; 4 Department of Pediatric Neurosciences, SRCC NH Children’s Hospital, Mumbai, India; 5 Department of Pediatric Neurosciences, Jaslok Hospital & Research Center, Mumbai, India; 6 Department of Pediatrics, Division of Infectious Diseases, Global Health Center, Children’s Hospital of Philadelphia, Philadelphia, PA USA; 7 Department of Pediatric Neurology, Faculty of Medicine, Hacettepe University, Ankara, Turkey

**Keywords:** Treatment, Antiviral agent, Subacute sclerosing panencephalitis, Measles complication, SSPE

## Abstract

**Purpose of the review:**

Subacute sclerosing panencephalitis (SSPE) is a rare, slowly progressive, and frequently fatal neurodegenerative disorder caused by measles virus. The risk of SSPE remains significant globally, with fluctuating incidence noted in in tandem with measles vaccine uptake. This review aims to explore the current global status of SSPE, its treatment, and preventive measures.

**Recent findings:**

An increase in measles cases have been reported in various parts of the world for different reasons related to the regional context of the outbreak. With reduction in measles vaccine doses since the onset of the COVID-19 pandemic, the future risk of SSPE can only accelerate. In recent years, subsequent cases of SSPE have been reported in the period following documented measles outbreaks in different settings. Concomitantly, there have been efforts to evaluate the efficacy of immunomodulatory, antiviral, and anti-seizure therapies that could ameliorate the devastating effects of this disease. This review elucidates on these approaches and their limitations, reasons for poor vaccine coverage in low- and middle-income countries, as well as the possible solutions to the prevention of measles and eventual avoidance of SSPE.

**Summary:**

Prevention of measles virus infection with the resultant sequelae would be the most effective strategy for the management of SSPE. This approach would be particularly important in low resource setting that currently bears the double burden of widespread communicable diseases and malnutrition.

## Introduction

Subacute sclerosing panencephalitis (SSPE) is a rare, slowly progressive, and frequently fatal neurodegenerative disorder caused by measles virus [1]. This disease usually occurs a median of 10 years after measles infection but latency period varies from 1 month to 27 years [1, 2•]. The worldwide incidence of SSPE is approximately 4–11 cases per 100,000 cases of measles [2•]. There is a geographical variation in the prevalence of SSPE with though the risk of SSPE remains significant globally, with fluctuating incidence noted in tandem with measles vaccine uptake [3, 4•, 5]. A review of studies in California between 1998 and 2015 showed a risk of SSPE of 1 in 1367 for children below 5 years and 1 in 609 for children less than 12 months at the time of measles disease [6]. In Germany, a population study between 2003 and 2009 showed a risk of SSPE of 1 in 1700 to 1 in 3300 after measles infection [7].

A study in South India reported an annual incidence of 4.3 cases per million for children below 20 years [8]. This high incidence was related to overcrowded living conditions and poor vaccination coverage. In developing countries like Pakistan, the risk is estimated as 10 cases per 1 million (5). In Papua New Guinea, following a large measles epidemic in 2002, a review of SSPE during the period between 2007 and 2009 reported an annual incidence of 54 cases per one million for those aged below 20 years (6). Four districts had an incidence of > 100 per million per year [9]. Population-based studies on the estimates of SSPE in Africa are lacking. Following measles epidemic in South Africa in 2009, 3–4 years later, children developed SSPE [7].

Children who contract measles before 5 years of age are at higher risk of developing SSPE later in childhood. Children with, or exposed to, human immunodeficiency virus infection who contract measles may be at increased risk of SSPE [10•]. The high incidence observed in developing countries is related to low rates of protective immunity to measles, frequent measles outbreaks, crowded living environments, and poor vaccination coverage. Other risk factors include poor socioeconomic status, malnutrition, low level of parental education, failure to receive vaccination, high number of siblings, and a high birth order [7–9, 10•].

## Pathophysiology

Mutations in the infecting measles virus have been thought to allow for establishment of a persistent infective state within the nervous system. Various pathophysiologic mechanisms for this persistent infection have been hypothesized, but none has been proven in the recent years. Typically, there is a history of measles illness with uneventful recovery, and several years later, the affected persons develop insidious onset of behavioral changes, neurocognitive impairment, and movement disorders followed by frank dementia. The illness is generally rapidly progressive, with death occurring between several months and 3 years following onset, and this is generally due to secondary complications [11]. Approximately one-fifth of cases of measles infection which do not present with a rash reports on SSPE without a previous history of measles are likely to result from those in apparent infections [5].

Subacute sclerosing panencephalitis is caused by particular mutations of the measles virus, characterized by the inability to produce infectious viral particles, neuro-pathogenicity in animal models as well as in humans, and the prolonged persistence in vivo over many years. The viral genome exhibits particular mutations referred to as biased hyper mutations, most notably in the M gene, followed by the F and H genes. There are consequential mutations of the M, F, and H proteins which are thought to account for the characteristic features of the measles virus which cause SSPE [12].

The pathogenesis of SSPE is yet to be illustrated fully; however, genetic studies have shown it to be caused by mutant wild strains of measles virus and not by the vaccine strains [3]. Many genotypes have been associated with the endemic circulation of the measles virus in certain geographical regions or are documented during an outbreak in a specific area. In a study in seven southern African countries during the epidemic of 2009 to 2010, the measles virus genotype detected was predominantly B3 [13]. The subsequent case series of children who developed SSPE showed, for the first time, that genotype B3 is linked to this disease [14]. Analyses of measles virus sequences in brain tissue samples obtained from children with SSPE have identified only wild-type measles virus, and the virus genotypes identified are consistent with the genotype of measles virus that circulated in the area where the patients lived and to which the affected children were exposed before the onset of SSPE [14]. Genetic studies support epidemiological evidence that the measles vaccine virus does not cause SSPE [14].

Recent evidence suggests that mutations that alter viral envelope glycoproteins, in particular the F protein, are responsible for the neuro-virulence of measles virus [15, 16]. The measles virus has been demonstrated to enter the brain during the primary infection. By remaining in cells, the virus evades the host’s immune response [15, 16]. Strong antiviral immune responses in the host are evidenced by high levels of specific antibodies in the blood and cerebrospinal fluid (CSF) and are a characteristic feature of this condition [17]. It is a possibility that SSPE is the result of a poor cellular immune response. This is demonstrated by the fact that SSPE is a more prevalent complication in younger patients exposed to the measles virus, most likely due to their immature immune system. There is evidence to suggest that patients who develop SSPE have a reduced cellular immune response and an elevated humoral immune response, which would prevent the patient from completely eradicating the virus [11].

The actual mechanism of CNS infection by the measles virus is not clear. However, protein F SLAM, Nectin, and probably others are postulated to play a role in viral entry into neurons where the measles virus undergoes mutations that allow for evasion of host immune response and continuous viral production without damaging neuronal cells [16]. Neuro-virulence of SSPE strains of the measles virus is likely due to the impaired expression of the M protein. Various studies have suggested that apoptosis of various cell types may contribute to the neuro-pathogenesis of measles virus infection in the human central nervous system, either as a direct effect of viral infection or of cytokine-mediated responses, resulting in oligodendroglial and neuronal cell death in SSPE [18].

## Treatment options

### Immunomodulatory drugs

#### Isoprinosine

Isoprinosine is the first drug that has been used in the treatment of SSPE. It is a synthetic combination of inosine and acedoben dimepranol with immunomodulatory and antiviral properties. Beside the immune-stimulatory effects like enhancing T cell proliferation and activity of natural killer cells and increasing pro-inflammatory cytokine levels, a direct effect on viral RNA levels has been demonstrated [19]. In a multinational study with 500 SSPE cases, Isoprinosine was found to have the widest usage among other treatment options in participant countries [20]; however, there are concerns about its availability in countries where SSPE is common due to its high cost [2•]. Isoprinosine’s beneficial effect on survival and neurological deficiencies has been achieved in one-third of cases of SSPE given at 50–100 mg/kg/day (maximum of 3 g/day) orally in three to five divided doses as a monotherapy or combined treatment with Interferons (IFN) [21]. It is usually well tolerated; however, transient nausea, hyperuricemia, and renal stones may occur and follow-up is required.

#### Interferons

Interferons (IFNs) are endogenous immunomodulatory molecules produced by immune cells and used in the treatment of SSPE since they have inhibitory effect on viral replication. It has been reported that intraventricular IFN‐α treatment combined with Isoprinosine induced remission or stabilization in 44–55% of SSPE cases [22]. However, long-term follow-up of these patients revealed that neurological deterioration occurs eventually and treatment-induced remission in SSPE appears to be temporary. It has been proposed that longer treatments with higher doses might sustain the treatment effect [23]. In a report comparing Isoprinosine with Isoprinosine plus intraventricular IFN-α combination, no difference was found in the improvement rate when Isoprinosine was administered alone [21]. Aseptic meningitides has been reported following intraventricular IFN treatment. Other adverse effects include fever, lethargy, and loss of appetite. Subcutaneous IFN-β (22 µg, 3 times per week) in combination with Isoprinosine might extend the survival and delay progression in SSPE [24]. The wide availability and easy applicability of IFN-β enhance its applicability.

#### Amantadine

Amantadine appeared to improve survival and was associated with sustained clinical improvement in a retrospective analyses of 38 SSPE patients [25]. A study which compared amantadine, Isoprinosine, and IFN-α showed that all three drugs have a relative ameliorative effect on the disease; however, Isoprinosine was four times more effective than amantadine and twice as effective as IFN-α [26].

#### Intravenous immunoglobulin

There are single case reports that indicate clinical improvement to various degree following intravenous immunoglobulin (IVIG) treatment in SSPE [27]. One retrospective cohort study reported temporary clinical improvement especially among patients who received IVIG therapy during the early stages of the disease [28].

#### Aprepitant

Measles virus uses cell surface receptors to spread among host cells during infection. Neruokinin-1 receptor, a cell surface receptor, has been shown to mediate the trans-synaptic transmission of measles virus. In a recent randomized double-blind placebo-controlled clinical study of aprepitant, a neurokinin-1 receptor antagonist, no clinical but a modest EEG improvement was observed in SSPE patients [29•].

### Antiviral agents

#### Ribavirin

Ribavirin is a nucleic acid analogue used in the treatment of RNA viral infections, primarily against hepatitis C and respiratory syncytial viruses in clinical practice. Ribavirin was found to inhibit replication of SSPE virus strains in in vitro studies and animal models [30]. Ribavirin has been used in SSPE patients intravenously or intrathecally in combination with interferon-alpha with and without Isoprinosine. Partial improvement in symptoms or slower progression has been reported in some cases. Intraventricular administration is preferred to maintain effective ribavirin concentrations in brain tissue. In a recent report, three SSPE patients were treated with continuous intra-ventricular infusion by subcutaneous implantable infusion pump to maintain CSF concentration and avoid frequent lumbar punctures [31].

#### Favipiravir and remdesivir

Favipiravir and remdesivir are nucleic acid analogues which interfere with viral RNA polymerase activity. Favipiravir was developed for the treatment of influenza virus and its efficacy against different RNA viruses including the SSPE strain has been reported [32, 33•]. The main challenge is maintenance of sufficient CSF concentration due to its oral administration; hence, further studies are needed in Favipiravir usage in SSPE. The therapeutic activity of remdesivir has been shown against several RNA viruses including measles virus in cell-based assays [34]. Animal models indicate that remdesivir can attain therapeutic levels in the brain in following intravenous administration [35].

### Anti-seizure medications

#### Carbamazepine

Carbamazepine (CBZ) plays an important role in the symptomatic treatment of SSPE. Although CBZ is known to exacerbate generalized myoclonus in epilepsy, it is paradoxically effective in the treatment of myoclonus in some patients with SSPE. The mechanism of action of CBZ on myoclonus of SSPE is not totally understood but may be related to the likely basal ganglia origin of the myoclonic activity [36–38]. Clobazam, levetiracetam, and valproate are other alternatives for the treatment of myoclonus and seizures in SSPE [30].

#### Ketogenic diet

Ketogenic diet is widely used to manage drug-resistant epilepsy with effects observed through inhibition of neuronal hyper-excitability. Ketogenic diet also has antioxidant and anti-inflammatory effects; therefore, it is of increasing interest in the management of other neurological diseases like Alzheimer disease, migraine, and motor neuron diseases [39]. In a previous report, ketogenic diet led to a temporary improvement on myoclonic jerks in SSPE [40]. Recently, a beneficial effect on clinical, cognitive function, and EEG findings was reported in an SSPE patient following use of ketogenic diet [41].

## Prevention of SSPE

### Vaccination

The incidence of SSPE is inversely related to immunization coverage as demonstrated in various studies across the world. In resource poor countries where malnutrition and exposure to other infectious diseases are common, the case fatality ratio for measles commonly rises to 5% but can be as high as 30% in refugee camps or in isolated immunologically naïve populations [9]. Although the number of measles deaths has declined progressively since year 2000, measles remains a leading cause of vaccine preventable deaths in children younger than 5 years in many regions in the world particularly Sub-Saharan Africa and South East Asia [42]. Infants born to unvaccinated mothers in an under vaccinated population are at higher risk of contracting measles even right after birth and therefore are at the highest risk for SSPE. Trans-placentally transmitted anti-measles antibodies can temporarily protect infants from measles infection. Successful immunization programs protect against SSPE and virology studies have shown that measles vaccine virus does not cause SSPE [43•]. A population study in Istanbul for the period 2002 and 2004 showed a risk of SSPE of 2 per million population and measles vaccination was found to be highly protective against SSPE [44].

### Vitamin A

Measles infection is affected by Vitamin A status especially in children below 2 years of age. Low serum vitamin A levels are associated with increased mortality from measles. A study done in Turkey by Gungor et al. [45] reports on children on follow up for SSPE between 2001 and 2010, and found that their serum alpha-tocopherol, betacarotene, retinol, ascorbic acid levels and erythrocyte and cerebrospinal fluid glutathione levels were all lower compared to the control groups.

#### Factors that hinder measles eradication in the developing world

The goal in eradication of any disease is to halt all transmission by extermination of the causative agent through surveillance and containment. This is certainly achievable in the case of measles given that there is only one antigenic type of measles virus which causes the disease and it does not survive outside of the human host. Measles infectivity is generally 4 days prior to and up to 4 days following development of the rash, which allows for possible identification of primary cases and isolation to prevent infection of non-immune contacts. Immunity against measles infection is life-long following administration of two vaccine doses [46•].

Despite all these factors that provide an opportunity for measles eradication, transmission continues in low- and middle-income countries (LMIC) as evidenced by data that shows that measles cases have continued to climb into 2019. Reported cases rose by 300 percent in the first 3 months of 2019, compared to the same period in 2018 with several outbreaks of measles also documented in high-income countries [47]. In the context of the coronavirus disease 2019 (COVID 19) pandemic, immunization monitoring systems have identified marked reductions in the number of doses of measles-containing vaccine that have been ordered and administered, compounding a preexisting problem and implying an expected increase in the cases of measles in 2020 and beyond [48].

Strategic containment and eradication of measles critically relies upon well-functioning national immunization programs and surveillance systems. Underfunding by government and donor agencies in LMIC has contributed to inadequate vaccine coverage. The global coverage with the first dose of measles vaccine has stalled at 85 percent, which falls below the 95 percent level needed to prevent outbreaks, while the second dose coverage, though increasing, currently stands at a suboptimal level of 67 percent [36, 37].

A myriad of factors contributes to ongoing transmission of measles in LMIC including poor management of vaccine supply chains allowing frequent dosage stock outs and inadequate supply especially in hard to reach areas. Paucity of healthcare centers in Africa in particular secondary to under investment in infrastructure development also contributes to low vaccination rates in general [49•].

Unreliable and inaccurate surveillance data on measles vaccination uptake from LMIC hinders appropriate planning for dose delivery and population specific strategies. Investment in maintenance of robust surveillance systems is required to overcome this hurdle [38].

Vaccine hesitancy has been present throughout the history of vaccines but in recent times has begun to play a more prominent role in reduction in measles vaccination uptake eroding some of the previous gains [46•, 50, 51]. Due to the interruption of transmission in high-income countries, measles cases are now rare and sporadic; hence, families fail to see the necessity to vaccinate their children. Misguided information regarding vaccinations has contributed to certain group of people not presenting children for measles vaccination resulting in sizeable populations of vulnerable children. A recent review confirms that unvaccinated individuals constitute the majority of the measles cases during outbreaks, the majority of whom were unvaccinated due to parental choice. Unvaccinated migrant populations also contribute a significant number and to incidents of measles outbreaks (imported measles) especially when coupled with sub-optimal vaccination rates in the population [50].

## Strategies to overcome barriers to measles eradication in low- and middle-income countries

Increased research effort towards the development of more successful immunization programs that leverage on context-specific approaches is needed in developing countries.

Accurate immunization-related education for the general public from primary school all the way to tertiary institutions including those that train healthcare workers is critical as these are the parents and practitioners of tomorrow.

Sustained engagement and education of the general public should be clearly supported by governments and other agencies as well as healthcare practitioners. Awareness creation should be intensified to inform concerned citizens about the urgent necessity of measles vaccination.

Government funding needs to be enhanced and focused towards provision of two doses of measles vaccine for all children. Already from 2001 to 2020, for every US$1 invested in measles vaccine, $58 were saved in future costs in 73 low-income and middle-income countries [46•].

Vaccine storage, distribution, handling and stock management, and monitoring systems need to be automated with a focus to ensure that vaccine availability in a viable form is maintained.

Surveillance systems within countries need to be appropriately funded and enforced to provide data which governments and donor agencies can rely on for effective planning and vaccine provision. Improved accessibility of the vaccine for migrant and pastoralist communities in developing countries in form of mobile health clinics coupled with mass sensitization and mop up campaigns would be useful. Community-based data collectors and local knowledge can help adapt public health programming to the local context and could aid disease eradication in at-risk populations [52•].

Measles is eradicable and vaccine-preventable. However, in recent years, re-emergence of measles infection-related cases and deaths has been observed, and the global surge in measles should be “a wake-up call” that was stated in The Lancet report published in 2019 [51].

## Conclusions

Sub-acute sclerosing panencephalitis (SSPE) remains a clinical condition associated with significant morbidity and mortality for which we have demonstrated the limitations of currently available treatment options that may delay but do not prevent the eventual demise of those affected by the disease. The risk of contracting SSPE is particularly significant for population living in low resource areas where availability of palliative treatments is not attainable. Populations in these regions are also faced with effects of malnutrition and the additional challenges of diseases such as HIV and tuberculosis which further complicate the use of potentially effective medications such as carbamazepine.

We have explored factors that have potentially contributed to the hindrance of measles eradication in LMICs which specifically revolved around the reduction in vaccine availability, access, and supply, resulting from multiple underlying reasons. Furthermore, this paper has highlighted strategies that can be implemented for measles eradication in LMICs which focused on ensuring attainment of high and complete measles vaccine coverage, which remains the best option in avoidance of SSPE in all settings and would be the most impactful for low resource areas.
